# Early‐Onset Neonatal Pityriasis Versicolor Associated With Maternal Infection: A Case Report From Uganda

**DOI:** 10.1002/ccr3.72762

**Published:** 2026-05-19

**Authors:** Abdisalam Ahmed Sandeyl, Bahari Yusuf, Yasin Ahmed H. Abshir, Abdullahi Ahmed Omar, Abdurahman Hussein Muhumed, Enass Ahmed Abdulgadir

**Affiliations:** ^1^ Internal Medicine Department Kampala International University Western Campus Ishaka‐Bushenyi Uganda; ^2^ Pediatrics and Child Health Department Kampala International University Western Campus Ishaka‐Bushenyi Uganda; ^3^ Dermatology Department Kampala International University Western Campus Ishaka‐Bushenyi Uganda

**Keywords:** early‐onset, Malassezia, maternal infection, neonate, pityriasis versicolor, Uganda

## Abstract

We report a case of early‐onset neonatal pityriasis versicolor (PV) in a term newborn associated with maternal infection. The mother had characteristic hypo‐ and hyperpigmented, finely scaly lesions over the neck and upper trunk prior to delivery. Within the first weeks of life, the neonate developed multiple hypopigmented, finely scaly macules involving the trunk and proximal thighs. The infant was otherwise clinically well, with no history of prematurity, neonatal intensive care unit admission, antibiotic exposure, or underlying medical illness. The clinical features were consistent with PV, and the diagnosis was confirmed by potassium hydroxide (KOH) microscopy demonstrating Malassezia species in both the mother and infant. Treatment with topical antifungal therapy resulted in clinical improvement. This case highlights that PV, although rare in neonates, can present early in life, particularly in the context of close maternal contact. Increased awareness of this entity may facilitate prompt diagnosis, prevent unnecessary investigations, and ensure appropriate management.

## Key Clinical Message

Neonatal pityriasis versicolor is rare, with fewer than 15 infant cases reported in the case‐report literature. It may present within the first weeks of life, particularly with active maternal infection. Recognition of its characteristic lesions and simple mycological confirmation allows prompt diagnosis and effective topical treatment, avoiding unnecessary investigations.

## Graphical abstract

Early‐onset neonatal pityriasis versicolor lesions.

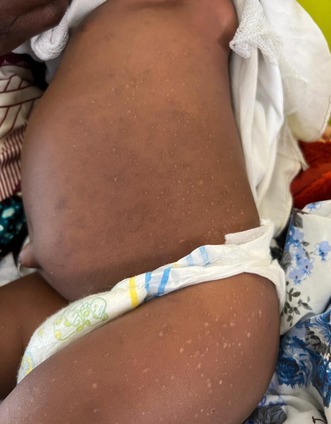

**Abbreviations**
KOH: potassium hydroxide
PV: pityriasis versicolor

## 1 Introduction

Pityriasis versicolor (PV) is a common superficial fungal infection caused by Malassezia species, lipophilic yeasts that constitute part of the normal cutaneous flora [1]. It typically affects adolescents and adults and presents as hypo‐ or hyperpigmented macules with fine scaling, most commonly involving the trunk and proximal extremities [2]. Predisposing factors include warm climates, increased sebaceous activity, immunosuppression, and close skin contact [3].

In neonates, PV is rare. Immaturity of sebaceous gland activity and reduced skin surface lipids in early life limit Malassezia proliferation [4]. Consequently, hypopigmented dermatoses in neonates are more commonly attributed to other conditions, and PV may be overlooked [5].

We report a case of early‐onset neonatal PV associated with maternal infection in a term newborn, highlighting this rare presentation and underscoring the importance of considering this diagnosis in neonates with compatible clinical features.

## 2 Case Presentation

### 2.1 Case History and Examination

A 29‐day‐old male neonate presented to Kisoro District Hospital with generalized skin discoloration noted during the early neonatal period. He was born at term via spontaneous vaginal delivery following an uncomplicated pregnancy, with a birth weight of 3.5 kg. There was no history of perinatal asphyxia, neonatal intensive care unit admission, postnatal complications, antibiotic or topical medication exposure. The infant was exclusively breastfed.

**FIGURE 1:**
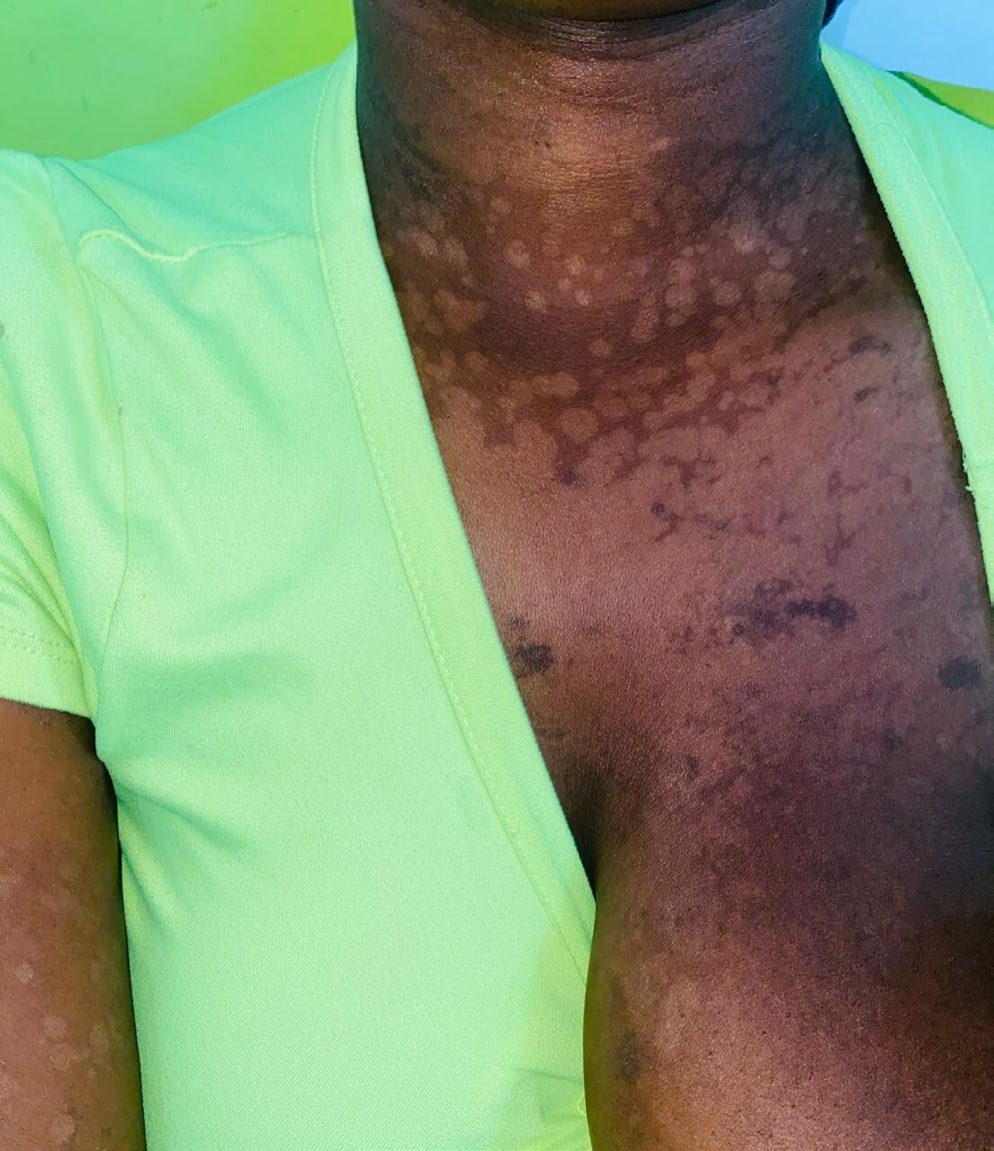
Maternal lesions of pityriasis versicolor. Hypo‐ and hyperpigmented, finely scaly macules and patches on the mother's neck, upper trunk, and arms, consistent with active pityriasis versicolor.

The mother reported a two‐month history of hypo‐ and hyperpigmented, finely scaly skin lesions involving the neck, anterior chest, and upper limbs, present prior to delivery and clinically consistent with PV (Figure 1). She described frequent close skin‐to‐skin contact with the infant after birth. Cutaneous lesions in the infant were first noted at two weeks of life as hypopigmented macules, which progressively increased in number. There were no associated systemic symptoms, including fever, irritability, poor feeding, or vomiting.

**FIGURE 2:**
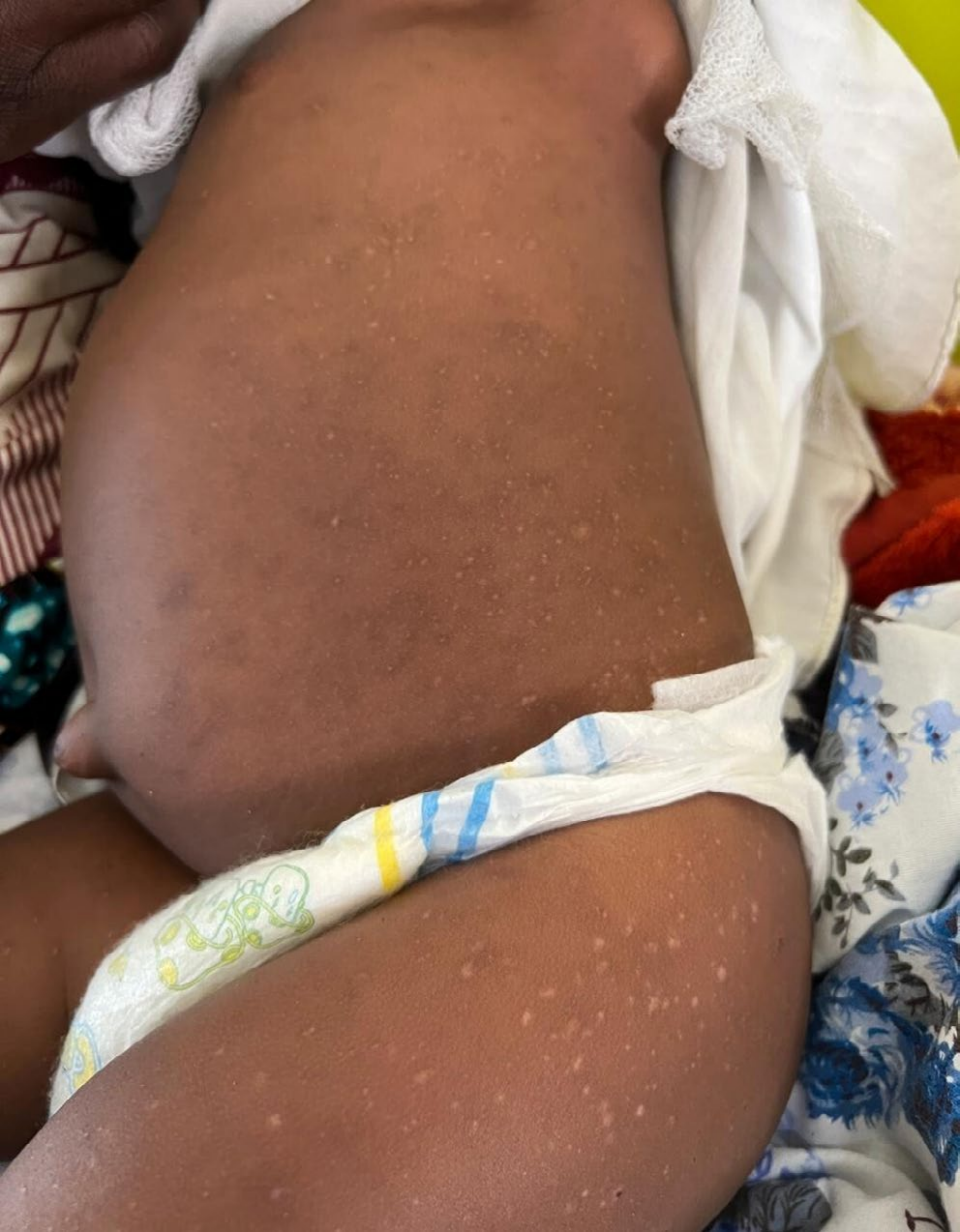
Neonatal lesions of pityriasis versicolor. Multiple well‐defined hypopigmented macules with fine superficial scaling on the trunk and proximal thighs of a 29‐day‐old infant, consistent with early‐onset pityriasis versicolor.

On examination, the infant was clinically well, afebrile, and hemodynamically stable. Dermatologic examination revealed multiple well‐defined hypopigmented macules and small patches, measuring approximately 2–8 mm, with fine superficial scaling, distributed over the trunk and proximal thighs, with partial coalescence in some areas (Figure 2). There was no erythema, vesiculation, pustulation, or mucosal involvement. The scalp, palms, soles, and diaper area were spared. The remainder of the systemic examination was unremarkable.

### 2.2 Investigations and Differential Diagnosis

KOH microscopy of skin scrapings obtained from the infant demonstrated short hyphae and spores consistent with Malassezia species, confirming the diagnosis of PV. KOH examination of the mother's lesions yielded similar findings. Maternal investigations, including complete blood count and HIV testing, were within normal limits.

Given the early neonatal onset, differential diagnoses considered included neonatal candidiasis, seborrheic dermatitis, pityriasis alba, pityriasis versicolor, and post‐inflammatory hypopigmentation. However, the absence of erythema or satellite pustules, lack of greasy scales or scalp involvement, characteristic lesion morphology, and positive mycological findings in both the mother and infant supported the diagnosis of neonatal PV.

### 2.3 Treatment and Outcome

Both the mother and infant were treated with topical clotrimazole 1% cream applied twice daily to the affected areas for four weeks, which was well tolerated. At six‐week follow‐up, the infant showed a favorable clinical response, with resolution of scaling and partial improvement of pigmentary changes. Repeat KOH microscopy of skin scrapings at follow‐up was negative. The infant remained clinically stable, with normal feeding and activity.

## 3 Discussion

### 3.1 Early‐Onset Neonatal Pityriasis Versicolor

Pityriasis versicolor (PV) is an infection caused by Malassezia species that typically affects adolescents and adults, with neonatal involvement being distinctly rare [1, 2]. The low frequency in neonates is thought to relate to reduced sebaceous gland activity and lower skin lipid content early in life, which limits fungal proliferation [4]. Consequently, PV is often not considered when evaluating pigmentary disorders in the neonatal period.

In this case, lesions appeared within the first two weeks of life, representing an unusually early presentation. A review of the literature indicates that fewer than 15 infant cases have been reported in the case‐report literature, with ages ranging from 3 weeks to 8 months [6, 7, 8, 9, 10, 11, 12, 13]. Recent data from a retrospective study of 415 children with PV found that 35% were under 6 months of age, with the youngest patient being 1 month and 15 days old [14]. These observations support the occurrence of PV in very young infants and highlight that it can manifest even during the neonatal period. Consequently, PV should not be excluded solely on the basis of age when clinical features are suggestive.

### 3.2 Role of Maternal Disease and Close Contact

A notable feature of this case is the presence of active maternal PV at the time of neonatal presentation. Although Malassezia species are part of the normal skin microbiota, symptomatic infection in neonates is uncommon, and the mechanisms underlying early disease remain incompletely understood [1, 15]. Close maternal–infant contact may facilitate early colonization, particularly in the presence of active maternal infection. Maternal infection has been reported in only two prior cases [8, 9], suggesting that maternal–infant transmission may be a rare but important contributor to neonatal PV.

Several factors may contribute to early colonization in neonates, including frequent skin‐to‐skin contact, local warmth, and occlusion, particularly during breastfeeding. These conditions may create a microenvironment favorable for Malassezia proliferation [3].

The apparent rarity of reported maternal infection in previous cases may reflect underreporting, as maternal examination was not consistently documented. This suggests that maternal colonization may be more common than recognized and highlights the importance of evaluating and reporting maternal skin findings in future cases.

### 3.3 Diagnostic and Management Considerations

The diagnosis of PV in neonates can be challenging due to overlap with more common neonatal dermatoses, including neonatal candidiasis, seborrheic dermatitis, pityriasis alba and post‐inflammatory hypopigmentation [5]. In this case, the characteristic morphology, fine superficial scaling, and KOH microscopy demonstrating Malassezia species allowed a confident diagnosis and exclusion of alternative conditions.

Other non‐invasive diagnostic modalities may support the diagnosis of PV. Wood's lamp examination can demonstrate a characteristic yellowish or golden fluorescence, while dermoscopy may reveal fine scaling and altered pigmentary patterns. Ultraviolet fluorescence‐based dermatoscopy has also been described as a useful adjunct. However, these tools may not be readily available in resource‐limited settings, making KOH microscopy a practical and accessible diagnostic method [3, 16].

There are no standardized treatment guidelines for neonatal PV. However, topical antifungal agents are considered safe and effective for superficial Malassezia infections and remain first‐line therapy [17]. The favorable response to topical clotrimazole in this infant supports its use in neonatal disease. Systemic antifungal therapy was not indicated. Concurrent treatment of the mother was undertaken to reduce ongoing exposure and potential recurrence.

A summary of reported cases of infantile PV under 1 year, including lesion distribution, maternal history and treatment is provided in Table 1.

**TABLE 1:** Reported cases of infantile pityriasis versicolor under 1 year of age.

Author/Year	Sex/Age	Region	Physical exam	Location	Family history	Past medical history	Delivery	Treatment
Congly (1984) [6]	Male/3 months	Canada	Erythematous scaly macules and patches	Dorsal upper arm, shoulders, upper back	Negative	Negative	Not available	Clotrimazole 1% solution, twice daily for 4 weeks
Di Silverio et al. (1995) [7]	Male/2 months	Italy	Hyper‐ and hypopigmented scaly macules	Cervical, scalp, face, upper chest	Negative	Negative	Normal vaginal delivery	Econazole 1% lotion (frequency and duration not reported)
Nanda et al. (1998) [8]	Male/3 weeks	India	Several hypopigmented macules	Forehead	Negative	Negative	Normal vaginal delivery	Clotrimazole 1% solution (frequency and duration not reported)
	Male/4 months	India	Hypopigmented scaly lesion	Neck, upper trunk, arms, face	Positive (Mother)	Negative	Not available	Clotrimazole 1% solution (frequency and duration not reported)
	Male/5 months	India	Light brown, scaly macules	Neck	Negative	Atopic dermatiti‐s	Not available	Clotrimazole 1% solution (frequency and duration not reported)
	Male/4 weeks	India	Hypopigmented scaly macules	Forehead	Negative	Not available	Normal vaginal delivery	Clotrimazole 1% solution (frequency and duration not reported)
	Female/5 weeks	India	Hypopigmented scaly macules	Face, forehead	Negative	Not available	Not available	Clotrimazole 1% solution (frequency and duration not reported)
Said et al. (2010) [9]	Male/3 months	Tunisia	Hypopigmented macules	Cervical, chest	Positive (Mother)	Negative	Not available	Topical antifungal (details not reported)
Jubert et al. (2015) [10]	Male/3 weeks	Spain	Hypopigmented macules and patches	Upper trunk, face, neck	Negative	Prematur‐e, LBW, TPN, ICU, antibiotic‐s	Not available	Intravenous fluconazole for 2 weeks (dose and interval not reported)
Abdollahimajd et al. (2019) [11]	Female/8 months	Iran	Hypopigmented macules	Lateral face, neck, upper back, chest	Negative	Negative	Normal vaginal delivery	Clotrimazole 1% lotion, twice daily for 4 weeks
	Female/4 months	Iran	Hypopigmented macules	Frontal area of face	Negative	Negative	Normal vaginal delivery	Clotrimazole 1% lotion, twice daily for 4 weeks
Almalki et al. (2023) [12]	Male/3 months	Pakista‐n	Hypopigmented scaly macules	Trunk	Negative	Negative	Normal vaginal delivery	Clotrimazole 1% solution, twice daily (duration not reported)
Zhou et al. (2024) [13]	Male/7 months	China	Round‐to‐oval hypopigmented macules and patches	Scalp	Not available	Not available	Not available	Terbinafine hydrochloride cream, twice daily for 6 weeks (concentration not reported)
	Male/8 months	China	Round‐to‐oval hypopigmented macules and patches	Scalp	Not available	Not available	Not available	Terbinafine hydrochloride cream, twice daily for 8 weeks (concentration not reported)
Present case	Male/29 days	Uganda	Multiple hypopigmented macules with fine superficial scaling	Trunk, proximal thighs	Positive (Mother)	Negative	Normal vaginal delivery	Clotrimazole 1% cream, twice daily for 4 weeks

*Note:* Summary of previously reported cases of pityriasis versicolor in infants younger than 1 year, including author and year, sex and age, geographic region, physical examination findings, lesion distribution, family history, past medical history, delivery details, and treatment.

Abbreviations: ICU, intensive care unit; LBW, low birth weight; TPN, total parenteral nutrition.

### 3.4 Clinical Implications

This case highlights the importance of considering PV in neonates presenting with hypopigmented, finely scaly lesions, particularly in the context of active maternal infection. Early recognition can facilitate appropriate topical treatment, avoid unnecessary investigations, and prevent misdiagnosis, contributing to improved neonatal dermatologic care.

## 4 Conclusion

Early‐onset neonatal PV is rare but can occur within the first weeks of life. This case, in the context of confirmed maternal infection, contributes to the limited body of reported infant cases under 1 year of age. It underscores the importance of considering PV in neonates with compatible hypopigmented, scaly lesions. Prompt recognition and treatment with topical antifungal therapy are effective and can prevent misdiagnosis and unnecessary interventions. Future reports should consider systematic evaluation and documentation of maternal skin findings to better understand potential transmission dynamics.

## Author Contributions

**Abdisalam Ahmed Sandeyl:** conceptualization, investigation, project administration, writing – original draft, writing – review and editing. **Bahari Yusuf:** conceptualization, resources, writing – review and editing. **Yasin Ahmed H. Abshir:** data curation, investigation. **Abdullahi Ahmed Omar:** investigation, writing – review and editing. **Abdurahman Hussein Muhumed:** investigation, writing – review and editing. **Enass Ahmed Abdulgadir:** resources, supervision, writing – review and editing.

## Funding

The authors have nothing to report.

## Consent

Written informed consent was obtained from the patient's parent for publication of this case report and any accompanying images.

## Conflicts of Interest

The authors declare no conflicts of interest.

## Data Availability

All data supporting the findings of this case report are contained within the article. No additional datasets were generated or analyzed.
